# Mode division multiplexing using an orbital angular momentum mode sorter and MIMO-DSP over a graded-index few-mode optical fibre

**DOI:** 10.1038/srep14931

**Published:** 2015-10-09

**Authors:** Hao Huang, Giovanni Milione, Martin P. J. Lavery, Guodong Xie, Yongxiong Ren, Yinwen Cao, Nisar Ahmed, Thien An Nguyen, Daniel A. Nolan, Ming-Jun Li, Moshe Tur, Robert R. Alfano, Alan E. Willner

**Affiliations:** 1 Department of Electrical Engineering, University of Southern California, Los Angeles, CA 90089 USA; 2 Institute for Ultrafast Spectroscopy and Lasers, CUNY City College, New York, NY 10031 USA; 3 Physics Department, CUNY City College, New York, NY 10031 USA; 4 Physics Department, CUNY Graduate Center, New York, NY 10016 USA; 5 New York State Center for Complex Light, New York, NY 10031 USA; 6 School of Engineering, University of Glasgow, Glasgow G12 8QQ, Scotland, UK; 7 Corning Incorporated, Sullivan Park, Corning, 14831 NY USA; 8 School of Electrical Engineering, Tel-Aviv University, Tel-Aviv, ISRAEL 69978.

## Abstract

Mode division multiplexing (MDM)– using a multimode optical fiber’s N spatial modes as data channels to transmit N independent data streams – has received interest as it can potentially increase optical fiber data transmission capacity N-times with respect to single mode optical fibers. Two challenges of MDM are (1) designing mode (de)multiplexers with high mode selectivity (2) designing mode (de)multiplexers without cascaded beam splitting’s 1/N insertion loss. One spatial mode basis that has received interest is that of orbital angular momentum (OAM) modes. In this paper, using a device referred to as an OAM mode sorter, we show that OAM modes can be (de)multiplexed over a multimode optical fiber with higher than −15 dB mode selectivity and without cascaded beam splitting’s 1/N insertion loss. As a proof of concept, the OAM modes of the LP_11_ mode group (OAM_−1,0_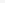 and OAM_+1,0_), each carrying 20-Gbit/s polarization division multiplexed and quadrature phase shift keyed data streams, are transmitted 5km over a graded-index, few-mode optical fibre. Channel crosstalk is mitigated using 4 × 4 multiple-input-multiple-output digital-signal-processing with <1.5 dB power penalties at a bit-error-rate of 2 × 10^−3^.

Mode division multiplexing (MDM) – using a multimode optical fiber’s N spatial modes as data channels to transmit N independent data streams – has received interest as it can potentially multiply optical fiber transmission capacity N-times with respect to single mode optical fibers (SMFs)^1^,^2^,^3^. Many spatial mode bases can be used for MDM. One basis is that of linearly polarized (LP) modes^4^,^5^,^6^,^7^. However, other bases can be used^8^,^9^. A basis that has received interest is that of OAM modes. In the paraxial approximation, a light field having an 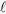-dependent and azimuthally varying phase given by 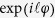 has an OAM of 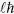 per photon along its propagation direction, where 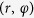 are cylindrical coordinates, 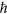 is the reduced Planck constant, and 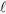 is an integer (“topological charge”)^10^. This was first realized for Laguerre-Gaussian modes^11^. In this paper, we refer to such light fields as OAM modes. OAM modes have been used for MDM with Tbit/s transmission capacity over free-space and optical fibres^12^,^13^. Regardless of spatial mode basis used, MDM is affected by mode coupling, resulting in channel crosstalk, which can, however, be mitigated using multiple-input-multiple-output-digital-signal-processing (MIMO-DSP)^4^,^5^,^6^.

Two challenges of MDM are (1) designing mode (de)multiplexers with high mode selectivity (2) designing mode (de)multiplexers without 1/N insertion loss of using cascaded beam splitters. When (de)multiplexing with high mode selectivity, mode dependent effects, such as, mode-dependent loss can be equalized and MIMO-DSP complexity can be reduced^14^,^15^,^16^. There are many mode (de)multiplexers that have overcome these challenges. For example, a mode selective photonic lantern, which comprises N symmetrically arranged, dissimilarly sized, and adiabatically tapered SMFs, has mode selectivity as high as −20 dB^17^,^18^,^19^,^20^. Another example is a free space device from CAILabs, which is based on multi-plane light conversion, and has mode selectivity as high as −23 dB^21^,^22^,^23^.

In this paper, using a device referred to as an OAM mode sorter^24^,^25^,^26^, we show that OAM modes can be (de)multiplexed over a multimode optical fiber with higher than -15 dB mode selectivity and without cascaded beam splitting’s 1/N insertion loss. As a proof of concept, the OAM modes of the LP_11_ mode group, each carrying 20-Gbit/s polarization division multiplexed (PDM) and quadrature phase shift keyed data streams, are transmitted 5 km over a graded-index, few-mode optical fibre. Channel crosstalk is mitigated using 4 × 4 MIMO-DSP with <1.5 dB power penalties at a 2 × 10^−3^ bit error rate (BER).

## OAM mode sorter

**Figure 1:**
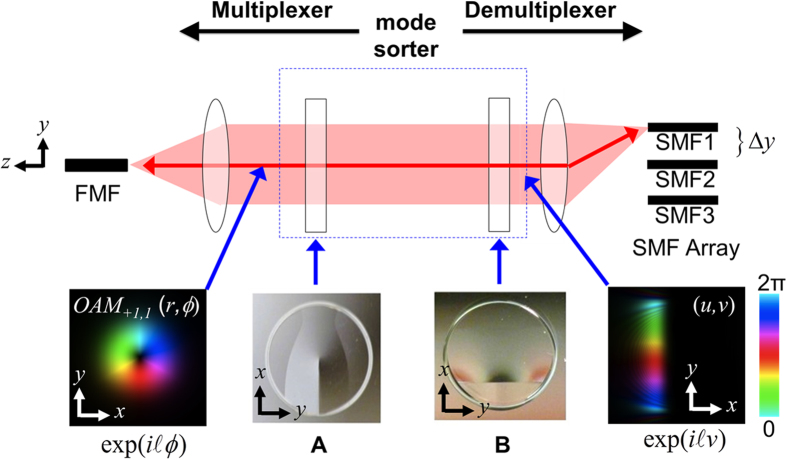
Concept of the OAM mode sorter. The OAM mode sorter functions as a mode multiplexer from right to left (SMF array to FMF), and functions as mode demultiplexer from left to right (FMF to SMF array). An OAM mode sorter consists of two custom refractive optical elements: (**A**) and (**B**).

A description of the OAM mode sorter’s principle of operation is schematically shown in Fig. 1^24^,^25^. The OAM mode sorter comprises two custom refractive optical elements (Inset A and B in Fig. 1) that carry out the geometric transformation 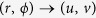, where 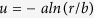 and 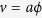, 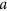 and 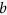 being experimental parameters^25^. When an OAM mode propagates through the OAM mode sorter, its 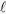-dependent and azimuthally varying phase, 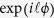, is transformed into an 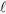-dependent and linearly varying phase, 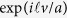, and its radially dependent intensity profile is transformed into an intensity profile elongated along 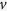. When focused by a lens, the intensity profile elongated along 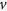 is focused to a spot elongated along 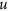 whose lateral position in the lens’s focal plane, due to the linearly varying phase, varies proportional to 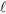. Effectively, using the OAM mode sorter, N multiplexed OAM modes each with a different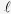, can be transformed into N laterally separated and elongated spots. Importantly, when the OAM mode sorter is used in the reverse direction, N laterally separated and elongated spots can generate N OAM modes that are spatially multiplexed. An SMF array is placed in the lens’s focal plane such that each elongated spot can be coupled to or generated from each SMF. A compound lens system (cylindrical lens, two convex lenses) shall be used to match the sizes, shapes, and lateral spacing of the elongated spots to the SMF array. The cylindrical lens can partially compensate the elongated spots’ shapes in turn improving light coupling to the SMF array. Mismatch between the compound lens system and the SMF array can result in the generation of OAM modes with asymmetric intensity profiles and multiple intensity rings. However, as each SMF addresses a unique OAM mode, N OAM modes can be (de)multiplexed with high mode selectivity, and, as the OAM mode sorter is a single device, N OAM modes can be (de)multiplexed without cascaded beam splitting’s 1/N insertion loss.

## Experiment

As a proof of concept, the OAM modes of the LP_11_ mode group (OAM_−1,0_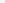 and OAM_+1,0_), each carrying 20-Gbit/s PDM-QPSK data streams, were transmitted 5km over a graded-index, few-mode optical fibre (See supplementary material section S.1). At 1550 nm, the few-mode optical fibre supported the LP_01_, LP_11_, LP_21_/LP_02_ mode groups (LP_21_ and LP_02_ mode groups are approximately degenerate). In general, modes in different mode groups experience significantly less mode coupling than modes in the same mode group^27^. Over 5km of the few-mode optical fibre, the OAM modes of the LP_01_ and LP_21_/LP_02_ mode groups experience negligible mode coupling with the two OAM modes of the LP_11_ mode group. Therefore, as a proof of concept, channel crosstalk can be mitigated using 4 × 4 MIMO-DSP (including two polarizations per mode). However, nothing limits the OAM mode sorter from simultaneously (de)multiplexing, for example, the OAM modes of the LP_01_ and LP_11_ mode groups.

**Figure 2 f2:**
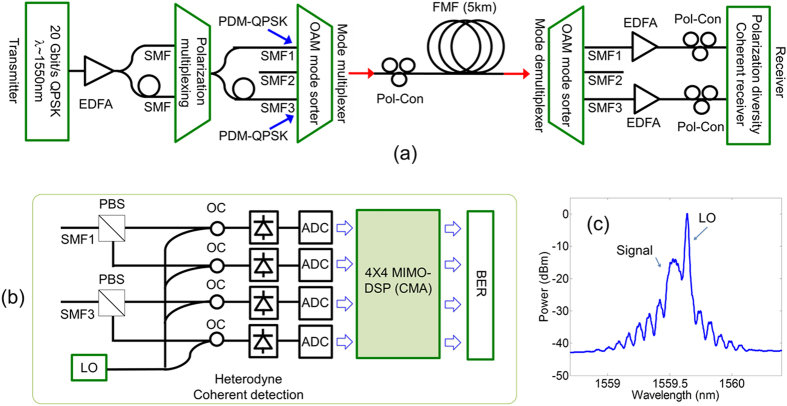
**(a)** Experimental setup as described in the text. (EDFA: erbium-doped fibre amplifier. SMF: single mode fibre. Pol-Con: polarization controller. PBS: polarization beam splitter. FMF: few-mode fibre.) **(b)** Experimental setup of the heterodyne coherent detection. (OC: optical coupler. LO: local oscillator. ADC: analogue-to-digital converter. MIMO-DSP: multiple-input-multiple-output digital signal processing. CMA: constant modulus algorithm). (**c**) Optical spectrum of the heterodyne detection - QPSK signal and the LO.

The experimental setup is shown in Fig. 2. The transmitter was as follows. A 20 Gbit/s QPSK data stream, being a 2^15^−1 length pseudorandom-binary-bit-sequence, was generated by modulating with an I-Q modulator an external cavity laser (ECL) output (λ ∼ 1550 nm). After amplification by an erbium doped fiber amplifier (EDFA), the data stream was decorrelated and polarization multiplexed via two SMFs. The resultant PDM-QPSK signal was again decorrelated via two more SMFs that were connected to a SMF array (~254 μm spacing). The light beam output from the SMF array was collimated and reshaped using compound lens system (cylindrical lens, two convex lenses), and then propagated in the reverse direction through the OAM sorter as described above.

**Figure 3 f3:**
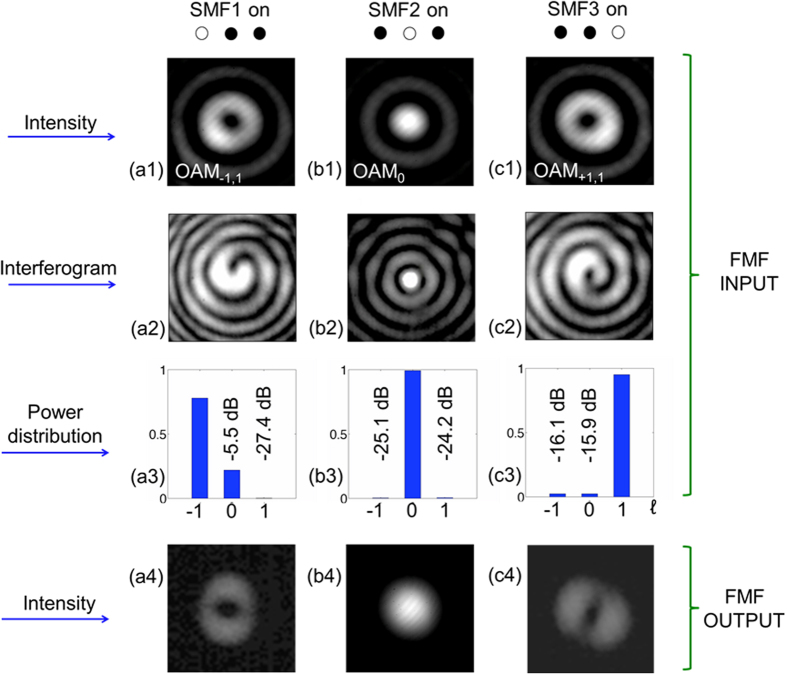
**(a1)–(c1)**: Intensity profiles of OAM modes as generated by the OAM mode sorter at the few mode optical fibre (FMF) input (**a1**) OAM_−1,0_. (**b1**) OAM_0,0_. (**c1**) OAM_+1,0_. **(a2)–(c2)**: “spiral” interferograms of each OAM mode (**a2**) OAM_−1,0_. (**b3**) OAM_0,0_. (**c4**) OAM_+1,0_. **(a3)–(c3)**: OAM spectra (“power distribution”) of each OAM mode (**a3**) OAM_−1,0_. (**b3**) OAM_0,0_. (**c3**) OAM_+1,0_. **(a4)–(c4)** Intensity profiles at the FMF output when coupling one OAM mode at a time at the FMF input (**a3**) SMF1 on - OAM_−1,0_. (**b3**) SMF2 on - OAM_0,0_. (**c3**) SMF3 on - OAM_+1,0_.

Intensity profiles of generated OAM_−1,0_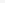, OAM_0,0_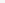, and OAM_+1,0_ modes were measured using an InGaAs camera (Fig. 3(a1),(b1),(c1)). As can be seen, the intensity profiles have multiple rings. This is due to mismatch in the mode shape of the fiber array output and the 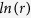 profile required for the transformer in reverse. This mismatch creates the same azimuthal (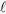) but higher radial (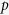) states, which are seen at the extra rings. Also, the intensity profiles are slightly asymmetric. This is also attributed to mismatch between the required and actual spacing of the SMF array. Effectively, a superposition of OAM modes is generated by each SMF. However, “spiral” interferograms of OAM_−1,0_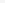, OAM_0,0_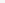, and OAM_+1,0_ modes, as created through their interference with an expanded Gaussian beam, qualitatively confirm the OAM modes’ correct azimuthally varying phases (Fig. 3(a2),(b2),(c2)). The mode selectivity of the OAM mode sorter was assessed by measuring the OAM spectrum of each generated OAM mode via a reflective, phase only, spatial light modulator^12^,^13^. Here, mode selectivity is defined as the amount of power generated in another OAM with respect to the desired OAM mode. As can be seen, the OAM mode sorter has mode selectivity higher than −15 dB.

The OAM_−1,0_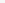 and OAM_+1,0_ modes, each carrying a PDM-QPSK signal, were then coupled to the few-mode optical fibre using an x−y−z translation stage and a 20X microscope objective. In a basis of OAM modes, including two orthogonal linear polarizations per mode, the LP_11_ mode group comprises OAM^x^_−1,0_, OAM^y^_−1,0_, OAM^x^_+1,0_, OAM^y^_+1,0_ (See supplementary material section S.1). Each OAM mode with a single polarisation serves as a data channel (Ch.) comprising four channels in total – Ch.1: OAM^x^_−1,0_, Ch.2: OAM^y^_−1,0_, Ch.3: OAM^x^_+1,0_, Ch.4: OAM^y^_+1,0_. A polarization paddle controller was used to bend the few-mode optical fibre near its input such that modes higher-order than the LP_11_ mode group, as generated due to imperfect coupling, experienced higher loss. The insertion loss of the few-mode optical fibre was −9 dB and is attributed to the multiple intensity rings of the generated OAM modes, and mismatch between the size of the light beam and the few-mode optical fibre core. This loss may be further reduced by an appropriate adjustment of the lens magnification. Intensity profiles at the output of the few-mode optical fibre when the OAM modes are coupled at its input, one at a time, are shown in Fig. 3(a4)–(c4). Note, the intensity profiles have a null point at their centre, qualitatively indicating that mode coupling between the LP_01_ and LP_11_ mode groups is negligible.

**Figure 4 f4:**
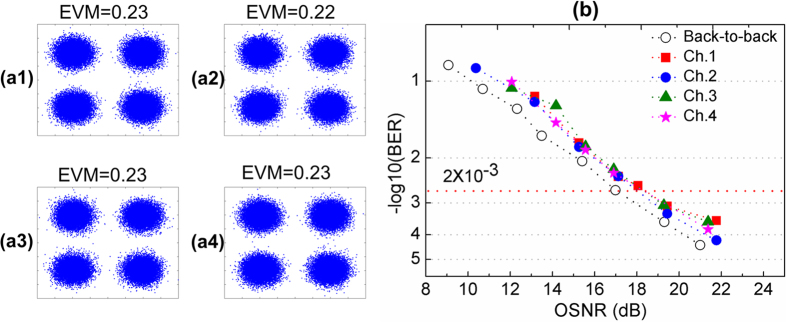
**(a1–a4)** Constellations of the 20-Gbit/s QPSK signals carried by each data channel (Ch.) after MIMO-DSP. (a1) Ch. 1 - OAM^x^_−1,0_, (a2) Ch. 2 - OAM^y^_−1,0_, (a3) Ch. 3 - OAM^x^_+1,0_, (a4) Ch. 4 - OAM^y^_+1,0_. (OSNR = 25.5dB) **(b)** BER vs OSNR curves for all Chs. and a back to back Ch.

The receiver was as follows. The few-mode optical fibre output was made to propagate through another OAM mode sorter using another x−y−z translation stage and a 20X microscope objective. The output was coupled into the SMFs of another SMF array corresponding to OAM_−1,0_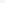 and OAM_+1,0_ modes resulting in two PDM-QPSK signals. The PDM-QPSK signals were amplified by an EDFA, and polarization demultiplexed and coherently detected (heterodyne) using a polarization diversity coherent receiver. The QPSK signals were mixed with a ~12 GHz frequency-offset local oscillator (LO) (Fig. 2(c)), converted to digital signals using four balanced detectors, and captured at 40 GSamples/s using real-time digital oscilloscopes, with an optical signal to noise ratio (OSNR) of ~25.5 dB, for offline MIMO-DSP (see supplementary material section S.2)^28^,^29^,^30^. MIMO-DSP comprised a constant modulus algorithm (CMA) and used 21 taps for each of the corresponding 16 finite-impulse-response (FIR) digital filters – the inverse channel matrix. The coefficients of each FIR filter were estimated after 10000 iterations of updating. A standard frequency offset estimation and carrier phase recovery were then implemented to recover the data. Figure 4(a1)–(a4, [Fig f2], [Fig f3], [Fig f4]) show constellation diagrams for each channel after MIMO-DSP. BER as a function of OSNR were measured for each channel and a back-to-back channel (Fig. 4(b)). As can be seen, all four channels reached a BER of <2 × 10^−3^, which is a threshold that can be corrected using forward error-correction coding. Power penalties of <1.5 dB were observed as compared to the back-to-back case at BER of 2 × 10^−3^.

## Discussion

In conclusion, using a device referred to as an OAM mode sorter, we showed that OAM modes can be (de)multiplexed over a multimode optical fiber with higher than -15 dB mode selectivity and without the 1/N insertion loss of using cascaded beam splitters. As a proof of concept, the two OAM modes of the LP_11_ mode group (OAM_−1,0_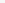 and OAM_+1,0_), each carrying 20-Gbit/s PDM-QPSK signals, comprising four channels, were transmitted over 5 km of a graded-index, few-mode optical fibre. 4 × 4 MIMO-DSP was used to mitigate the effects of mode coupling. Power penalties of <1.5 dB were observed at a BER of 2 × 10^−3^.

As a proof of concept, we only (de)multiplexed the two OAM modes of the LP_11_ mode group. However, there is no limitation on the OAM mode sorter in simultaneously (de)multiplexing the LP_01_ and LP_11_ mode groups. Over the same length of few-mode optical fibre, this would require additional 2X2 MIMO-DSP to mitigate the effects of polarization coupling between the two polarization of the LP_01_ mode group. In its current form, the OAM mode sorter can address the LP_01_ (OAM_0,0_), LP_11_ (OAM_+1,0_ and OAM_−1,0_) and LP_21_ (OAM_+2,0_ and OAM_−2,0_) mode groups. Scaling the OAM mode sorter to more mode groups requires an optical design that can address the radial index (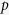) of spatial modes in higher-order mode groups (e.g. LP_02_, etc.). This has been experimentally investigated in^31^. However, the OAM mode sorter may be advantageous when used with few-mode optical fibres that exclusively support OAM modes without the need to address the radial index^13^.

The OAM mode sorter has been used in other research areas. For example, the OAM sorter has been used as an interface between path and OAM entanglement for high-dimensional quantum information^32^. Also, the OAM sorter has been used for high-dimensional quantum cryptography^33^. As such, it may be of interest to investigate the use of the OAM mode sorter to transmit both classical and quantum information over a few mode optical fibre^34^.

## Method

### Using the OAM mode sorter as a multiplexer

Data channels are connected to an SMF array with a physical spacing of 254 μm between two neighboring SMFs. The SMF array is placed in the focal plane of a lens combination with an effective focal length of ~655 mm. This lens combination converts the SMF array outputs into Gaussian waves each with a different phase tilt. These beams then pass though the mode sorter, which performs a geometrical transformation from Cartesian coordinates to log-polar coordinates. Each input beam with a different tilt phase is transformed into a specific OAM beam having a ring-shaped beam profile and a helical phase. Applying all the input beams with distinct tilt angles at the same time results in generation of multiple OAM beams propagating collinearly.

### Using the OAM mode sorter as a demultiplexer

Multiplexed OAM beams undergo a geometrical transformation from log-polar coordinates to Cartesian coordinates when passed though the mode sorter. The helical phase front and the ring shaped intensity of OAM mode are then transformed to a linearly changing phase front and a rectangular shaped intensity. A lens combination follows the mode sorter to focus the transformed beams such that, in the focal plane, each input OAM mode is mapped to a focused spot at a different lateral position. In this manner, each mode can be coupled into a different SMF of a fibre array.

## Additional Information

**How to cite this article**: Huang, H. *et al*. Mode division multiplexing using an orbital angular momentum mode sorter and MIMO-DSP over a graded-index few-mode optical fibre. *Sci. Rep*. **5**, 14931; doi: 10.1038/srep14931 (2015).

## Supplementary Material

Supplementary Information
