# Supplementary material for: Mode division multiplexing using an orbital angular momentum mode sorter and MIMO-DSP over a graded-index few-mode optical fibre
Source: Sci Rep. 2015 Oct 9;5:14931. doi: 10.1038/srep14931 (PMC4598738; doi:10.1038/srep14931)
Supplement: Supplementary Information [file srep14931-s1.pdf]

Supplementary

Mode division multiplexing using an orbital angular momentum mode sorter and MIMO-DSP over a graded-index few-mode optical fibre

Hao Huang[1,*], Giovanni Milione[2,3,4,5,*], Martin P. J. Lavery[6,*], Guodong Xie[1], Yongxiong Ren[1], Yinwen Cao[1], Nisar Ahmed[1], Thien An Nguyen[2], Daniel A. Nolan[5,7], Ming-Jun Li[7], Moshe Tur[8], Robert R. Alfano[2,3,4,5], and Alan E. Willner[1,*]

1. *Department of Electrical Engineering, University of Southern California, Los Angeles, CA 90089 USA*
2. *Institute for Ultrafast Spectroscopy and Lasers, CUNY City College, New York, New York, NY 10031 USA*
3. *Physics Department, CUNY City College, New York, NY 10031 USA*
4. *Physics Department, CUNY Graduate Center, New York, NY 10016 USA*
5. *New York State Center for Complex Light, New York, NY 10031 USA*
6. *School of Engineering, University of Glasgow, Glasgow G12 8QQ, Scotland, UK*
7. *Corning Incorporated, Sullivan Park, Corning, 14831 NY USA*
8. *School of Electrical Engineering, Tel-Aviv University, Tel-Aviv, ISRAEL 69978*

Corresponding emails: haoh@usc.edu, willner@usc.edu

S.1 Mutual relations of the OAM modes and the LP modes

Under the weakly guiding approximation, vector modes in a fibre can be described as [35]:

$$\begin{aligned} EH^{o}_{\ell-1,m}(r,\phi) &= f_{\ell m}(r)(\cos(\ell\phi)\hat{x}+\sin(\ell\phi)\hat{y}) \\ EH^{e}_{\ell-1,m}(r,\phi) &= f_{\ell m}(r)(-\sin(\ell\phi)\hat{x}+\cos(\ell\phi)\hat{y}) \\ EH^{o}_{\ell+1,m}(r,\phi) &= f_{\ell m}(r)(\cos(\ell\phi)\hat{x}-\sin(\ell\phi)\hat{y}) \\ EH^{e}_{\ell+1,m}(r,\phi) &= f_{\ell m}(r)(\sin(\ell\phi)\hat{x}+\cos(\ell\phi)\hat{y}) \end{aligned} \quad (1)$$

where (r,ϕ) are the cylindrical coordinates, $f_{\ell m}(r)$ are the solutions to the radial part of the wave

equation (ℓ= 0, 1, 2, …; m = 1, 2, …), and $\hat{x}$ and $\hat{y}$ are the Cartesian unit vectors for horizontal and vertical polarisation, respectively. Without loss of generality, the $\exp(i\beta_{\ell,m}z)$ and $\exp(i\omega t)$ dependence of each spatial mode was suppressed for brevity, where z is the propagation direction of light, $\beta_{\ell,m}$ is the propagation constant of each spatial mode, ω is the frequency of light, and t is time.

Another basis is the basis of LP modes. LP modes have been referred to as “pseudo modes”, because they are linear combinations of vector modes. LP modes are given by the following equations:

$$\begin{aligned}\hat{x}LP^{e}_{\ell,m} &= EH^{o}_{\ell-1,m} + HE^{o}_{\ell+1,m}\\ \hat{y}LP^{e}_{\ell,m} &= EH^{e}_{\ell-1,m} + HE^{e}_{\ell+1,m}\\ \hat{x}LP^{o}_{\ell,m} &= EH^{e}_{\ell-1,m} - HE^{e}_{\ell+1,m}\\ \hat{y}LP^{o}_{\ell,m} &= EH^{o}_{\ell-1,m} - HE^{o}_{\ell+1,m}\end{aligned} \tag{2}$$

where:

$$\begin{aligned}LP^{e}_{\ell,m}(r,\phi) &= f_{\ell m}(r)\cos(\ell\phi)\\ LP^{o}_{\ell,m}(r,\phi) &= f_{\ell m}(r)\sin(\ell\phi)\end{aligned} \tag{3}$$

The third mode basis is the basis of OAM modes. OAM modes have an azimuthal phase dependence of $\exp(i\ell\phi)$ [10]. Using Euler's identity, $\exp(i\ell\phi) = \cos(\ell\phi) + i\sin(\ell\phi)$, it can be shown that:

$$\begin{aligned}OAM_{\pm\ell,m} &= LP^{e}_{\ell,m} \pm iLP^{o}_{\ell,m}\\ &= f_{\ell m}(r)\exp(i\ell\phi)\end{aligned} \tag{4}$$

In terms of the vector modes, the OAM modes are given by the equations:

$$\hat{x}OAM_{\pm\ell,m} = EH^{o}_{\ell-1,m} \pm iEH^{e}_{\ell-1,m} + HE^{o}_{\ell+1,m} \pm iHE^{e}_{\ell+1,m}$$
$$\hat{y}OAM_{\pm\ell,m} = EH^{o}_{\ell-1,m} \pm iEH^{e}_{\ell-1,m} - HE^{o}_{\ell+1,m} \mp iHE^{e}_{\ell+1,m} \quad (5)$$

As seen in the above equations, a linear polarised OAM mode is a linear combination of all of the vector modes. Analogous to the LP modes, linearly polarised OAM modes are “pseudo modes”. As it is the vector modes that exhibit differing propagation constants, upon propagation through a multimode optical fibre, an OAM mode will experience what can be referred to as “modal quadrefringence” [36].

Spatial modes can be grouped into mode groups. Mode groups are determined based on the propagation constants. The propagation constants of the modes in the same mode group are more similar than those in different mode groups. As a result, modes in different mode groups experience less mode coupling than modes in the same mode group. For example, the LP11 mode group is composed of $TM_{0,1}$, $TE_{0,1}$, $HE^{o}_{2,1}$ and $HE^{e}_{2,1}$ vector modes. In the basis of OAM modes, the OAM modes of the LP11 mode group are given by the equations:

$$\hat{x}OAM_{\pm1,0} = TM_{0,1} \pm iTE_{0,1} + HE^{o}_{2,1} \pm iHE^{e}_{2,1}$$
$$\hat{y}OAM_{\pm1,0} = TM_{0,1} \pm iTE_{0,1} - HE^{o}_{2,1} \mp iHE^{e}_{2,1} \quad (6)$$

The mutual relationships among the three different mode bases are illustrated in Fig.S1.

S.2 Heterodyne detection and MIMO-DSP

In this experiment, heterodyne detection is used to demodulate the signal. After demultiplexing, each channel is combined with a local oscillator (LO) using a 3-dB optical coupler and detected by a photodiode (PD). The four LOs are derived from the same narrow linewidth laser, the wavelength of which is set to ~12 GHz away from the signal wavelength. After O/E conversion, the four signals

from the four PDs are simultaneously sampled by a 4-channel real time scope at a sampling rate of 40 GS/s on each channel, and then the signals are recorded for offline digital signal processing. The offline processing procedures is depicted in Fig.S1. Each of the four sequences is converted to the frequency domain and then band-pass filtered, followed by a 12-GHz frequency shift to the baseband. Next, the signals are converted back to time domain and down-sampled to 2 samples per symbol.

The MIMO DSP is used to mitigate the mode coupling effects [4, 6, 14-16]. MIMO-DSP functions as a linear equaliser that estimates the channel matrix by searching for the coefficients of each finite-impulse response (FIR) filter. For a 4×4 MIMO system, the equaliser includes 16 FIR filters. The output of the equaliser can be expressed as [6]:

$$y_j = \sum_i \mathbf{w}_{ij} * \mathbf{x}_i \tag{7}$$

where $\mathbf{w}_{ij}$ (i, j=1,2,3,4) is the coefficient vector of the FIR filter with a vector length of N, $\mathbf{x}_i$ is the input signal vector of the ith channel, and y_i is the output of the FIR filter. The notation * represents the convolution between two vectors. All the FIR coefficients are initialised as identity, and then updated until the coefficients are convergent based on the constant modulus algorithm (CMA) [28-30]:

$$\mathbf{w}_{ij}(k+1) = \mathbf{w}_{ij}(k) + u \cdot e_i \cdot y_i \cdot \mathbf{x}_i^{conj} \tag{8}$$

where u is the step size, $e_i = P_{ref} - |y_i|^2$ is the error signal of the adaptive estimation, and P_{ref} is the normalised reference power of the QPSK signal. The number of taps in each filter is set to 21, which is enough to cover the differential time delays among each data sequence. The obtained FIR filter coefficients are used to equalise the crosstalk among the QPSK channels carried on four OAM modes based on equation (7). After equalisation, the FFT-based frequency offset estimation and carrier phase recovery algorithms are applied to recover the signal constellations.

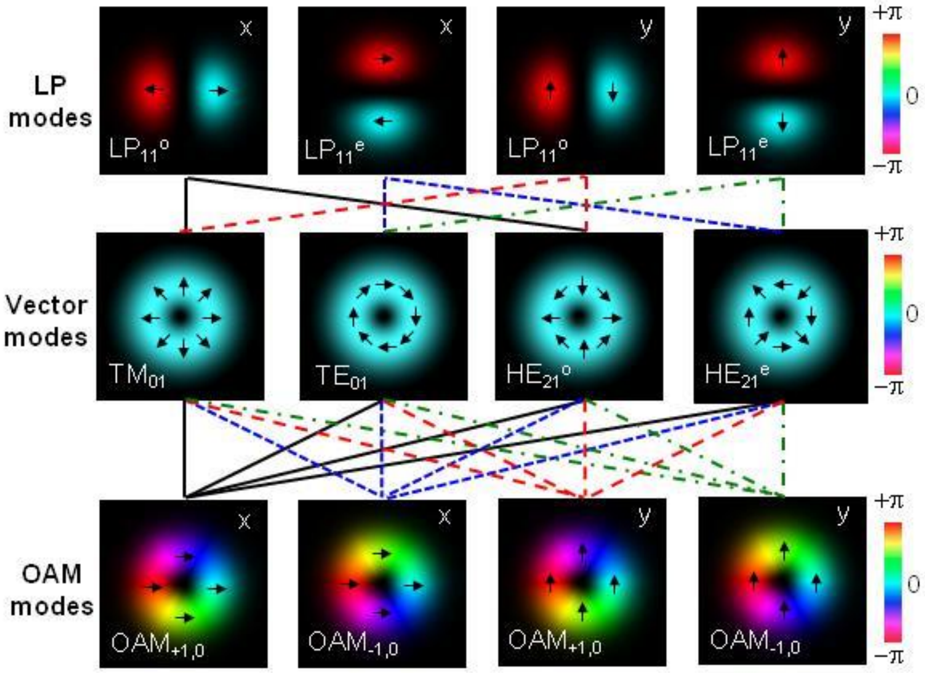

Fig.S1 Concept diagram showing three different mode bases (LP modes, vector modes and linearly polarized OAM modes) and their mutual relations for the LP11 mode group. (The colours illustrate the transverse phase profiles. The black arrows indicate the polarisation direction. “x” or “y” on the top-right corner also indicates the polarisation direction).

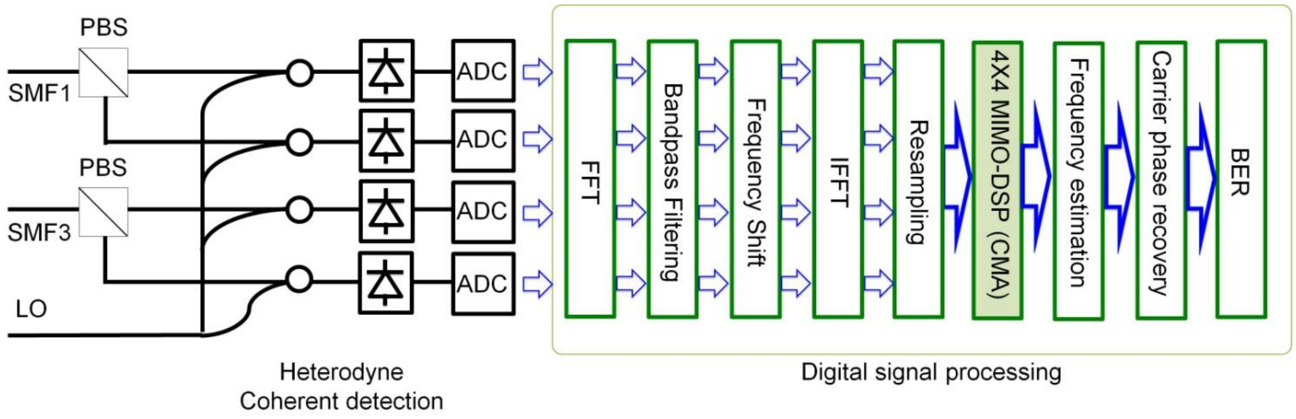

Fig.S2. Heterodyne coherent receiver and the MIMO DSP procedures. (LO: local oscillator. PBS: polarisation beam splitter. ADC: analogue-to-digital converter. FFT: fast Fourier transform. IFFT: inverse fast Fourier transform. MIMO: multiple input multiple output. CMA: constant modulus algorithm. BER: bit-error-rate.)
